# Factors related to mental health problems in nursing students: a multicenter study

**DOI:** 10.15649/cuidarte.3296

**Published:** 2024-05-16

**Authors:** Diana Carolina Tiga-Loza, Ligia Betty Arboleda de Pérez, María Ángela Ramírez-Cruz, Rocío de Diego Cordero

**Affiliations:** 1 Universidad de Santander, Bucaramanga, Colombia. Universidad Manuela Beltrán, Bucaramanga, Colombia. E-mail: dia.tiga@mail.udes.edu.co Universidad de Santander Universidad de Santander Bucaramanga Colombia dia.tiga@mail.udes.edu.co; 2 Universidad de Santander, Bucaramanga, Colombia. E-mail: li.arboleda@mail.udes.edu.co Universidad de Santander Universidad de Santander Bucaramanga Colombia li.arboleda@mail.udes.edu.co; 3 SENA, Seccional Bucaramanga, Colombia. E-mail: maramirezcr@sena.edu.co SENA Bucaramanga Colombia maramirezcr@sena.edu.co; 4 Universidad de Sevilla, España. E-mail: rdediego2@us.es Universidad de Sevilla Universidad de Sevilla Spain rdediego2@us.es

**Keywords:** Mental Health, Students, Nursing, COVID-19, Patient Health Questionnaire, Mental Disorders, Salud Mental, Estudiantes de Enfermería, COVID-19, Cuestionario de Salud del Paciente, Trastornos Mentales, Saúde Mental, Estudantes de Enfermagem, COVID-19, Questionário de Saúde do Paciente, Transtornos Mentais

## Abstract

**Introduction:**

The COVID-19 pandemic has brought consequences to the mental health of the undergraduate population of nursing programs.

**Objective:**

To identify the factors associated with mental health disturbances among university students in nursing programs during the COVID-19 pandemic.

**Materials and Methods:**

A multicenter, cross-sectional study was conducted during the COVID-19 pandemic among students enrolled in nursing programs at two Colombian universities and one Spanish university. An online sociodemographic, economic, and personal survey was administered along with the GHQ-12, the Family APGAR, the MOS Social Support Survey, and the IES-R for posttraumatic stress. The prevalence of mental health disturbances and their differences according to the characteristics of the students were estimated; prevalence ratios were also obtained.

**Results:**

Of the 302 students, a prevalence of clinically significant mental was found in 61.92%, family dysfunction in 61.58%, and low social support in 9.33%. In addition, 44.46% had posttraumatic stress symptoms, 52.65% had economic difficulties, 54.61% had academic difficulties, and 69.87% had personal difficulties. These mental disturbances were frequent in the presence of moderate family dysfunction (PR=1.77 CI95%=1.15;2.73), difficulty in paying for food (PR=1.35 CI95%=1.09;1.67), a breakup with a partner (PR=1.27 CI95%=1.02;1.59) and clinically relevant posttraumatic stress symptoms (PR=1.69 CI95%=1.28;2.24).

**Discussion:**

Psychological distress and its related factors found in nursing students agree with other findings in the literature.

**Conclusion:**

A significant proportion of nursing students were affected in their mental health during the pandemic, demonstrating the need for systematic, continuous, and comprehensive strategies by educational institutions.

## Resumen

**Introducción:**

La pandemia de COVID-19 ha traído consecuencias en la salud mental de la población universitaria de enfermería.

**Objetivo:**

Identificar los factores relacionados con las alteraciones en la salud mental de universitarios de los programas de enfermería durante la pandemia por COVID-19.

**Materiales y Métodos:**

Estudio transversal multicéntrico, en estudiantes de enfermería de dos universidades colombianas y una española durante la pandemia por COVID-19. Se aplicó una encuesta por internet sobre aspectos sociodemográficos, económicos y personales, junto con los cuestionarios de salud mental GHQ-12, APGAR familiar, apoyo social MOS y estrés postraumático IES-R. Se estimó la prevalencia de alteraciones de la salud mental y sus diferencias según las características de los estudiantes, también se obtuvieron razones de prevalencia.

**Resultados:**

En 302 estudiantes se encontró una prevalencia de alteraciones mentales de importancia clínica de 61,92%, de disfunción familiar en 61,58%, con bajo apoyo social en 9,33%; además, presentaron síntomas de estrés postraumático (44,46%), sufrieron dificultades económicas (52,65%), académicas (54,61%) y personales (69,87%). Adicionalmente, estas alteraciones fueron más prevalentes ante una disfuncionalidad familiar moderada (RP=1,77 IC95%=1,15;2,73), ante la dificultad para pagar alimentos (RP=1,35 IC95%=1,09;1,67), la pérdida de la pareja (RP=1,27 IC95%=1,02;1,59) y ante síntomas de estrés postraumático (RP=1,69 IC95%=1,28;2,24).

**Discusión:**

El sufrimiento psicológico y sus factores relacionados encontrados en los estudiantes de enfermería concuerdan con otros hallazgos en la literatura.

**Conclusión:**

Una importante proporción de estudiantes de enfermería estuvieron afectados en su salud mental durante la pandemia, mostrando una necesidad de estrategias sistemáticas, continuas e integrales por parte de las instituciones formativas.

## Resumo

**Introdujo:**

A pandemia de COVID-19 trouxe consequéncias para a saúde mental da populado universitária de enfermagem.

**Objetivo:**

Identificar os fatores relacionados as alterares na saúde mental de estudantes universitários dos cursos de enfermagem durante a pandemia de COVID-19.

**Materiais e Métodos:**

Estudo transversal multicéntrico em estudantes de enfermagem de duas universidades colombianas e uma espanhola durante a pandemia de COVID-19. Foi aplicado um inquérito online sobre aspectos sociodemográficos, económicos e pessoais, juntamente com os questionários GHQ- 12 de saúde mental, APGAR familiar, MOS de apoio social e IES-R de stress pós-traumático. Também foram estimadas as prevaléncias de transtornos de saúde mental e suas diferen^as de acordo com as características dos estudantes.

**Resultados:**

Em 302 estudantes foi encontrada uma prevaléncia de transtornos mentais clinicamente importantes de 61,92%, de disfun^áo familiar em 61,58%, com baixo apoio social em 9,33%; além disso, apresentaram sintomas de estresse pós-traumático (44,46%), sofreram dificuldades económicas (52,65%), académicas (54,61%) e pessoais (69,87%). Além disso, essas alterares foram mais prevalentes diante da disfun^áo familiar moderada (RP=1,77 IC 95%=1,15;2,73), diante da dificuldade para pagar a alimentado (RP=1,35 IC 95%=1,09;1,67), a perda do companheiro (RP=1,27 IC 95%=1,02;1,59) e sintomas de estresse pós-traumático (RP=1,69 IC 95%=1,28;2,24).

**Discussáo:**

O sofrimento psíquico e seus fatores relacionados encontrados em estudantes de enfermagem concordam com outros achados da literatura.

**Conclusáo:**

Uma propor^áo significativa de estudantes de enfermagem foi afetada em sua saúde mental durante a pandemia, mostrando a necessidade de estratégias sistemáticas, continuas e abrangentes por parte das instituyes formadoras.

## Introduction

The COVID-19 pandemic, an unexpected health emergency that threatened human life, was considered a traumatic event on a global scale, with negative effects both on mental health and on the structure of personal, family, and community life. The pandemic resulted in a deterioration of the social fabric due to psychological distress caused by fear, infodemic, feelings of helplessness, and unmet basic needs accompanied by economic insecurity. The effects of these situations can be as varied as they are immediate, including the development of posttraumatic stress symptoms, depression, anxiety, inappropriate use of or dependence on alcohol or other substances^1^.

According to the World Health Organization (WHO), mental health disturbances are those cognitive, emotional, and behavioral disorders of clinical relevance that affect one in eight people worldwide, most commonly anxiety and depression, which have increased significantly as a result of the COVID-19 pandemic^2^.

Posttraumatic stress disorder (PTSD) is characterized by manifestations of severe anxiety that can lead to disability following exposure to any event that results in psychological trauma. During the COVID-19 pandemic, approximately 15% of nursing students in China were reported to have PTSD- related symptoms^3^; similarly, female university students in Italy were the most affected by PTSD^4^.

On the other hand, Li et al., who studied more than 6,000 nursing students in China, found an overall prevalence of anxiety of 34.97% and depression in 40.22% of the students^3^. In addition, approximately 25% of nursing students in Colorado (USA) expressed moderate to extremely high levels of negative emotional states, and 23.8% of students were rated within the range of clinical concern for the presence of posttraumatic stress disorder^5^.

The education sector, among other sectors, provided continuity to the activities programmed during the pandemic, using technological tools and platforms as an alternative for emergency distance learning. This required a sudden adaptation to digital media, which was described by teachers and students as a negative experience^6^. In addition to the accelerated and unstructured adaptation to distance learning, students and teachers faced personal, family, and work situations due to the pandemic that had a profound impact on physical and mental health.

In this sense, some factors have been reported to contribute to the occurrence of anxiety and depression in nursing students, such as family economic situation and social support. Those who reported low financial status were more likely to experience these disorders than those who reported good family financial status. They were also at higher risk for anxiety, depression, and PTSD when social support was low compared to students with high levels of social support^5^.

Having clear evidence of the impact on the mental health of undergraduate nursing students, who will be the future caregivers for the mental health of others, is considered of great importance. These disturbances should be evaluated comprehensively in the light ofthe changes in social, educational, housing, economic, and family dynamics, particularly for Spanish-speaking students, and with a differential perspective between countries with differing income levels such as Colombia and Spain. This evidence can be obtained using instruments for each of these dimensions, which can contribute to mental health care among students and future health emergencies.

Considering the above, the objective was to identify factors related to mental health changes in nursing students of two universities in Colombia and one in Spain during the COVID-19 pandemic.

## Materials and Methods

### Study design and population

The study followed a cross-sectional analytical or correlational multicenter design. It was conducted in two private universities in Bucaramanga, Colombia, with 9-10 semester nursing programs, and one public university in Seville, Spain, with a 4-year nursing program.

Among the criteria for inclusion were being a student enrolled and active in a professional nursing program or degree, aged 18+, having access to technological devices such as cell phones, computers, or tablets, and having an Internet connection. Students with a history of mental health problems or who did not answer more than 20% of the questions were excluded. The calculated sample size was 284 participants, for an estimated prevalence of mental condition of 25%, with a margin of error of a=0.05 and p=0.20^5^.

### Variables and data collection

Given that the aim was to comprehensively understand mental health disturbances and their relationship with sociodemographic, family, economic, social, and educational aspects, several measurement instruments were used through an online form that took approximately 30 minutes to complete.

The dependent variable was manifestations of mental health disturbances and was assessed using the General Health Questionnaire (GHQ-12). This instrument was used as a screening tool to identify clinically significant emotional disorders. The GHQ-12 consists of 12 items, 6 of which are measured on a 4-point Likert scale for positive responses (0=more so than usual, 1=same as usual, 2=less so than usual, and 3=much less than usual) and 6 for negative responses (0=not at all, 1=not more than usual; 2=rather more than usual; 3=much more than usual). The total score was calculated from the sum of the ordinal scores (0123), and a score greater than or equal to 12 was used as the cut-off point for significant disturbances^7^. This instrument has been validated for Colombia and Spain with overall Cronbach's alphas of 0.9 and 0.86, respectively, and takes approximately 5 minutes to complete^8^^, ^^9^.

Some sociodemographic and contextual characteristics were evaluated as independent variables, such as age, sex, socioeconomic status, housing conditions, employment status of students and their parents, job loss, difficulty paying for college, or living expenses. The presence of stressful situations such as failing classes, drop in GPA, and illness or death in the family during the pandemic was also assessed. For this component, the estimated completion time was 5 minutes.

The Family APGAR with 5 questions (Cronbach's alpha 0.819)^10^^, ^^11^, with cut-off points <10=normal functioning family, 10-12=mild dysfunction, 13-16=moderate dysfunction, and >16 points=severe dysfunction^(12)^, was used to determine students' perceptions of how their families function. This short instrument takes 2 minutes to complete.

In addition, the students' perceptions of the social support they receive were assessed using the Medical Outcomes Study (MOS) Social Support Survey Instrument, with Cronbach's alphas ranging from 0.921 to 0.736 in the Spanish version of the instrument^13^^, ^^14^. This questionnaire consists of 20 items; the first item asks about the number of people in the social network, and the other 19 items refer to the dimensions of emotional/informational support, tangible support, positive social interaction, and affectionate support. Social support was assessed using a 5-point Likert frequency scale (1= none of the time, 2= a little of the time, 3= some of the time, 4=most of the time, 5=all of the time), where the total score and the score by dimension are obtained by summing the scores; a score of less than 57 points represents low social support^15^. The estimated time to complete the instrument was 10 minutes.

Finally, the 22-item Impact of Event Scale-Revised (IES-R)^16^^, ^^18^, with a Cronbach's alpha of 0.9616 and a Likert scale ranging from 0 (never) to 4 (very often), was used to assess students' perceived posttraumatic stress due to the pandemic. For the analysis of posttraumatic stress with the IES-R, the established cut-off point was 25 points for clinically relevant stress and over 43 points for severe posttraumatic stress^19^. The estimated time to complete the instrument was 10 minutes.

### Data analysis

The variables were described by absolute and relative frequencies in percentages or by measures of central tendency and dispersion after verifying the normality of the distribution. According to the variable type, Pearson's chi-squared test, Fisher's exact test, Mann-Whitney U test, and Student's t-test were used to assess differences in mental health disturbances related to economic, personal, family, and health difficulties. In addition, the variables with a statistical significance level of less than 0.20 were included in a generalized linear regression model (GLM) using the Poisson log-link function to obtain the prevalence ratios of mental health disturbances^20^. The statistical software STATA, version 17, was used. All data collected are freely accessible for consultation on Mendeley Data^21^.

### Ethical considerations

All participants completed an oral informed consent form, which emphasized that participation was anonymous and voluntary. Due to the anonymous nature of the participation, there was no individual feedback on the results, but the results were shared as a group, and each academic program informed students of the mental health support offered by the universities. The study was approved by the Institutional Ethics Committee of Universidad de Santander, Meeting Minute No. 020 of May 20, 2021.

## Results

Between June and November 2021, 302 surveys met the inclusion criteria. Of these, 75 (24.83%) were completed by students from Spain and 227 (75.16%) from Colombia. The sample consisted mainly of female students (87.75%) with an average age of 22 years and a medium socioeconomic status (49.33%). In addition, 71.52% lived with their parents, and 50.99% had a stable romantic partner. Students reported experiencing economic (52.65%), academic (54.6%), and personal (69.87%) difficulties during the COVID-19 pandemic (Table 1). It was found that 187 students manifested clinically significant mental health disturbance, with a prevalence of 61.92% on the General Health Questionnaire GHQ-12, whose mean score was 13.8 (SD: 6.7). There was no statistical difference in prevalence of these disturbances between these two countries (58.67% in Spain and 63.00% in Colombia, p = 0.503), nor in mean GHQ-12 scores (13.52±5.4 in Spain and 13.93±7.04 in Colombia).

It was found that the prevalence of mental health disturbances was increased by 20% in those who had no partner or a casual partner (PR=1.20; 95%CI: 1.00, 1.4) and in those who had financial difficulties, especially in paying for food (PR=1.48; 95%CI: 1.26, 1.75), paying for housing (PR=1.46; 95%CI: 1.22, 1.75), and paying for internet (PR=1.22; 95%CI: 1.00, 1.48) compared to students who did not have these difficulties.

Similarly, the prevalence of mental health disturbances increased by 59% for students who broke up with their partner (PR=1.59; 95%CI: 1.28, 1.97), by 41% for those who reported conflicts with their family (PR=1.41; 95%CI: 1.17, 1.71), by 22% for those who became ill with COVID-19 (PR=1.22; 95%CI: 1.00, 1.49), and by 48% for those who had to be hospitalized (PR=1.48; 95%CI: 1.15, 1.92). We also found academic factors that increased the likelihood of reporting mental health disturbances, such as difficulty communicating with peers (PR=1.38; 95%CI:1.08, 1.76) or getting the connection equipment for taking online classes (PR=1.28; 95%CI1.02, 1.60).

**Table 1:** Characteristics of nursing students according to the occurrence of mental health disturbances, Bucaramanga-Colombia and Sevilla-Spain, 2021

Characteristics	Total %(n) (302)	No mental health disturbances %(n) (115)	Mental health disturbances %(n) (187)	p-value*	Prevalence ratio (95% CI)
Sex Male Female	12.25 (37) 87.75 (265)	11.30 (13) 88.70 (102)	12.83 (24) 87.17 (163)	0.694	1 1.14 (0.60; 2.14)
Age, median (min-max)	22 (20-24)	22 (20-24)	21 (20-24)	0.1988**	0.98(0.96; 1.00)
Socioeconomic status Low Medium High	47.68 (144) 49.33 (149) 2.98 (9)	46.08 (53) 48.69 (56) 5.21 (6)	48.86 (91) 49.73(93) 1.60 (3)	0.218	1 0.99 (0.83; 1.18) 0.52 (0.21; 1.34)
Home country Colombia Spain	75.16 (227) 24.83 (75)	37.00 (84) 41.33 (31)	63.00 (143) 58.67 (44)	0.296	1 0.93 (0.75; 1.15)
People you live with Friends Parents Relatives Alone	3.64 (11) 71.52 (216) 15.56 (47) 9.27 (28)	5.22 (6) 69.57 (80) 17.39(20) 7.83 (9)	2.67 (5) 72.73 (136) 14.44 (27) 10.16 (19)	0.536	1 1.39 (0.72; 2.67) 1.26 (0.63; 2.53) 1.49 (0.74; 3)
Partner Stable partner No partner or a casual partner	50.99 (154) 49.00 (148)	58.26 (67) 41.73 (48)	46.52 (87) 53.47 (100)	0.048	1 1.20 (1.00; 1.43)
Maintenance source Partner support Parents Themselves	5.30 (16) 76.49 (231) 18.21 (55)	6.96 (8) 73.91 (85) 19.13 (22)	4.28 (8) 78.07 (146) 17.65 (33)	0.546	1 1.26 (0.77; 2.09) 1.2 (0.70; 2.05)
Financial difficulties Paying for college Paying for food Paying for household Paying for transportation Paying for Internet service Job loss Death of financial supporters	52.65 (159) 40.38 (105) 20.23 (52) 15.57 (38) 27.06 (69) 30.92 (77) 27.20 (65) 28.85 (73)	46.09 (53) 38.24 (39) 8.33 (8) 6.45 (6) 22.45 (22) 23.96 (23) 23.16 (22) 26.00 (26)	56.68 (106) 41.77 (66) 27.33 (44) 21.19 (32) 29.94 (47) 35.29 (54) 29.86 (43) 30.76 (47)	0.073 0.570 1.06 <0.001 1.48 0.002 1.46 0.19 1.15 0.060 1.22 0.254 1.13 0.418 1.09	1.18 (0.98; 1.41) (0.87; 0.29) (1.26; 1.75) (1.22; 1.75) (0.94; 1.40) (1.00; 1.48) (0.92; 1.41) (0.89; 1.35)
Personal difficulties Death of a relative or someone close Serious illness of a family member Student with COVID-19 Illness that disabled or required Hospitalization Relationship breakup Conflicts with romantic partner Conflicts with family Conflicts at work	69.87 (211) 75.46 (203) 50.38 (131) 37.92 (91) 8.63 (17) 14.95 (32) 14.95 (32) 28.94 (68) 9.64 (19)	60.00 (69) 75.25 (76) 44.55 (45) 30.53 (29) 3.61 (3) 3.53 (3) 11.11 (10) 16.67 (15) 7.87 (7)	75.94 (142) 75.60 (127) 54.09 (86) 42.76 (62) 12.28 (14) 16.38 (19) 17.75 (22) 36.55 (53) 11.11 (12)	0.003 1.36 0.949 1.00 0.134 1.16 0.056 1.22 0.032 1.48 0.004 1.59 0.179 1.22 0.001 1.41 0.442 1.17	(1.08; 1.71) (0.81; 1.25) (0.95; 1.41) (1.00; 1.49) (1.15; 1.92) (1.28; 1.97) (0.94; 1.60) (1.17; 1.71) (0.55; 3.89)
Academic difficulties Failing classes Difficulty communicating with peers Difficulty communicating with teachers Difficulty concentrating on studying Difficulty getting the connection equipment for taking online classes	54.6 (165) 9.00 (18) 12.44 (26) 11.71 (24) 43.39 (105) 23.39 (51)	50.4 (58) 6.82 (6) 6.90 (6) 8.14 (7) 37.50 (36) 16.65 (15)	57.2 (104) 10.71 (12) 16.39 (20) 14.29 (17) 47.26 (69) 28.13 (36)	0.250 1.11 0.339 1.21 0.040 1.38 0.177 1.25 0.134 1.17 0.049 1.28	(1.93; 1.33) (0.85; 1.73) (1.08; 1.76) (0.94; 1.67) (0.95; 1.43) (1.02; 1.60)

* p-value estimated using Pearson's chi-squared test or Fisher's exact test; ** Mann-Whitney U test

It is noteworthy that 61.58% of the students had some degree of family dysfunction, 9.27% had low social support, and 44.40% had clinically relevant posttraumatic stress symptoms (Table 2). In addition, an association was observed between mental health disturbances and family function, social support, and posttraumatic stress symptoms (Table 2). As the Family APGAR scores increased, indicating greater family dysfunction, the prevalence of mental health disturbances increased. Lower mean social support scores, both globally and in each dimension, were also found among those who reported mental health disturbances, with a 30% increase in prevalence among those who reported low social support (PR=1.30 95% CI 1.05;1.62).

**Table 2:** Family function, social support, and posttraumatic stress symptoms according to the occurrence of mental health disturbances in nursing students, Bucaramanga-Colombia and Seville-Spain, 2021

Characteristics	Total %(n) (302)	No mental health disturbances %(n) (115)	Mental health disturbances %(n) (187)	p-value	Prevalence ratio (95% CI)	p-value
Family function-APGAR, median (minmax) Normal (<10 points), %(n) Mild dysfunction (10-12 points), %(n) Moderate dysfunction (13 - 16 points), %(n) Severe dysfunction (>16 puntos), %(n)	15 (11-18) 38.41 (116) 39.74 (120) 7.95 (24) 13.91 (42)	17 (1-18) 51.30 (59) 35.65 (41) 3.48 (4) 9.57 (11)	14 (3-18) 30.48 (57) 42.25 (79) 10.70 (20) 16.58 (31)	<0.001* 0.001**	0.96 (0.94; 0.98) 1 1.34 (1.07; 1.68) 1.70 (3.31; 2.19) 1.50 (1.15; 1.95)	0.001 0.011 <0.001 0.002
MOS Social Support Survey, mean (SD) Emotional, mean (SD) Tangible, mean (SD) Positive social interaction, mean (SD) Affectionate, mean (SD) With social support (>57 points), %(n) Low social support (<57 points), %(n)	79.7 (17,1) 33.3 (7.5) 16.7 (3.8) 16.9 (3.7) 12.7 (2.8) 90.73 (274) 9.27 (28)	85 (15.5) 35.7 (6.6) 17.8 (3.5) 18 (3.3) 13.5 (2,5) 94.78 (109) 5.22 (6)	76.4 (17.2) 31.9 (7.7) 16 (3.8) 16.3 (3.7) 12.2 (2.9) 88.24 (165) 11.76 (22)	<0.001† <0.001† <0.001† <0.001† <0.001† 0.041**	0.96 (0.94; 0.98) 0.98 (0.97; 0.99) 0.96 (0.94; 0.98) 0.96 (0.94; 0.98) 0.95 (0.92; 0.97) 1 1.30 (1.05;1.62)	0.001 <0.001 <0.001 <0.001 <0.001 0.016
Posttraumatic stress - IES-R, mean (SD) Normal (<24 points), %(n) Low risk (24-32 points), %(n) Likely risk (33-36 points), %(n) High risk (> 36 points), %(n) No clinically relevant symptoms (<32), %(n) With clinically relevant symptoms (>33), %(n)	29.3 (23) 48.13 (116) 7.47 (18) 4.98 (12) 39.42 (95) 55.60 (134) 44.40 (107)	16.7 (19.1) 73.26 (63) 4.65 (4) 3.49 (3) 18.60 (16) 77.91 (67) 22.09(19)	36.3 (22) 34.19 (53) 9.03 (14) 5.81 (9) 50.97 (79) 43.23 (67) 56.77 (88)	<0.001† <0.001** <0.001**	1.01 (1.01; 1.02) 1 1.7 (1.2; 2.3) 1.6 (1.1; 2.4) 1.8 (1.5; 2.3) 1 1.6 (1.36; 2)	<0.001 0.001 0.011 <0.001 <0.001

* Mann-Whitney U test, ** Chi-squared test/Fisher's exact test, tStudent's t-test, SD= Standard Deviation, MOS = Medical Outcomes Study, IES-R= Impact of Event Scale-Revised.

Next, it was observed that the mean score of the perceived stress scale was significantly higher in students with mental health disturbances during the pandemic (p<0.001), and the prevalence of these disturbances increased by 60% (PR=1.60) in those who manifested clinically relevant symptoms for posttraumatic stress (p<0.011).

**Figure 1:**
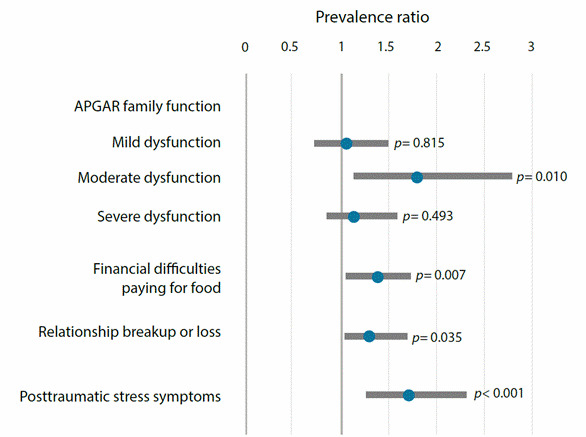
Adjusted prevalence ratios of mental health disturbances in nursing students, Bucaramanga-Colombia and Seville-Spain, 2021

*Note: The comparison category for family function was normal function; in the case of financial difficulties and relationship breakup or loss, these were compared with those who did not report them. Those with a score of 33 or higher on the IES-R were considered to have posttraumatic stress syndrome*

Finally, in the multiple regression model, the prevalence of mental health disturbances increased among students with moderate family dysfunction (PR=1.77 95%CI =1.15;2.73), difficulties paying for food (PR=1.35 95%CI =1.09;1.67), relationship breakup or loss (PR=1.27 95%CI =1.02;1.59), or clinically relevant posttraumatic stress symptoms (PR=1.69 95%CI =1.28;2.24) (Figure 1).

In this regard, during the COVID-19 pandemic, nursing students showed relevant mental health disturbances. The most affected were those who reported personal, family, economic, academic, and health difficulties, such as not having a partner, losing or breaking up with a partner, family conflicts, low social support, getting sick from COVID-19 or being hospitalized, problems paying for food, housing, and Internet, problems communicating with peers, and getting equipment to connect to virtual classes. Similarly, more than half of the students who presented mental health disturbances had symptoms of clinically relevant posttraumatic stress. No differences were found in the prevalence of mental health disturbances between Colombian and Spanish students.

## Discussion

A relevant finding of this study was that 61.92% of the nursing students showed symptoms of clinically important mental health disturbances during the COVID-19 pandemic. This prevalence was similar between Colombian (63.00%) and Spanish (58.67%) students. These results are consistent with those found in three European countries, where the prevalence of mental disorders such as depression was estimated at 59.1% of Spanish students, 34.5% of Albanian students, and 21.8% of Greek students^22^.

Similarly, Saudi nursing students reported 43.3% depression, 37.2% anxiety, and 30.9% stress as a result of the pandemic^23^. A review of 17 articles on the mental health of nursing students found that the most common problem during the pandemic was depression (52%), followed by anxiety (41%), stress (30%), and sleep disturbances (27%)^24^. Similarly, the results of the present study seem to be in line with those found in Turkey, where it was found that 71.5% of nursing students reported that they were at risk for mental health problems25. However, this estimate is superior because cutoff points were derived from the mean scores for each question, with > 2 points on the GHQ-12 scale considered as risk^25^.

Healthcare professions, including nursing, are characterized by a competitive environment, sensitivity to people's health, and dense theoretical content and practice with significant time commitment. Changes in contexts, study habits, and ability to concentrate can lead to frustration and anxiety, such as, for example, moving from studying in a university setting and groups to studying alone or under the watchful eye of parents, attending clinical rotations in fear of contracting COVID-19, feeling burdened by the possibility of infecting their relatives or having to find learning alternatives when clinic rotations with patients are restricted^26^.

In addition, this study found that 44.40% of nursing students had clinically relevant posttraumatic stress symptoms, with a mean score of 29.3 (SD:23) on the IES-R. These results are higher than those reported in Filipino students (mean 19.5; SD: 13.12)^27^ and close to the mean of 22.8 points in nursing students in the United States, where a high risk of posttraumatic stress was found in 22.7% of participants^28^. In contrast, in Spain, the prevalence of posttraumatic stress was 20.7%^29^. In contrast, 39.42% of the students in the present research were at high risk for posttraumatic stress. This finding should indicate the importance of mental health interventions conducted by educational institutions, even though the risk of contagion has decreased.

It is worth noting that 71.52% of the students in this study lived with their parents, and 78.15% reported some family dysfunction. A study in Portugal found that 88.2% of nursing students started living with their parents because ofthe lockdowns^30^. As a result of the pandemic, students living alone had to move back in with their families, which may have changed family dynamics and functioning as lockdowns, limited independence, and even feelings of being overwhelmed by household chores and university work converged.

The present study also demonstrated the economic difficulties experienced by nursing students, reported by 52.65% of them, and an increase in the prevalence of mental health disturbances in 35% of those who reported difficulties in paying for food (PR=1.35 95% CI = 1.09;1.67). In addition, there was a higher prevalence of mental health disturbances among those who reported difficulty paying for Internet and housing, vital necessities for a student during the pandemic. Also, Portuguese students mentioned having less money to cover expenses^30^. Similarly, another study in Portugal showed an association between home economics, psychological well-being, and coping among nursing students (p=0.024), as those who had a reduction in household income had lower scores on sociability (p=0.008), control of self and events (p=0.003), and self-esteem (p=0.042)^31^.

In the personal domain, students who did not have a stable partner or who lost or broke up with their partner also had a higher prevalence of mental health disturbances. Similarly, those who scored higher on the MOS Social Support Scale had a 30% increase in the prevalence of mental health disturbances. An Israeli study found an association between social support and academic self-efficacy in nursing students^32^. Social interaction difficulties caused by confinement may have affected the ability to receive help from peers and the university, which may have increased anxiety and loneliness. Therefore, when social support is limited, schools and universities should provide sufficient resources for developing meaningful coping mechanisms and strategies for psychological support and self-care^33^. Similarly, teachers should be trained to recognize mental health disturbances and encourage the search for support^33^^, ^^34^, as well as training programs to develop flexible and participatory strategies to promote social support networks.

It is important that nursing education institutions and programs promote posttraumatic psychological strengthening, resilience, and a sense of coherence so that students who have experienced the pandemic can prepare for their transition into professional practice^34^^, ^^35^.

Strengths of the present study include the use of validated instruments, the participation of multiple educational centers in two countries, and the comparisons made to identify factors associated with a higher prevalence of mental health disturbances. Some limitations should also be recognized, such as the anonymous participation of students and the participation of only three universities, which may limit the generalizability of the results. However, nursing students from other universities may have the same problem, especially if their socioeconomic and family characteristics are similar to those presented in the discussion. It should be noted that some students could not access the survey due to internet connection problems or because they did not want to complete the survey. Therefore, it is important to use short surveys and short versions of the scales.

## Conclusion

Although healthy young people are considered a low-risk population for COVID-19, the mental suffering and personal, economic, family, and educational difficulties of students, in this case nursing students, during the health emergency are evident.

This study shows that some students may be more likely to have mental health problems. Those who have family dysfunction or difficulty paying for food, who have experienced a breakup with their partner, or who have symptoms of posttraumatic stress disorder may be more likely to have mental health disturbances.

In addition, the results of this study support the emerging need for intervention and monitoring of modifiable factors that influence mental health disturbances in post-pandemic nursing students. Therefore, university welfare and support services should develop sustained strategies that address emotional-psychological distress with a focus on individual vulnerabilities and needs, as well as having a plan of action that addresses future collective threats in this regard.
